# A model to study the inhibition of nsP2B-nsP3 protease of dengue virus with imidazole, oxazole, triazole thiadiazole, and thiazolidine based scaffolds

**DOI:** 10.1016/j.heliyon.2019.e02124

**Published:** 2019-08-01

**Authors:** Vijay Kumar Vishvakarma, Nidhi Shukla, Reetu, Kamlesh Kumari, Rajan Patel, Prashant Singh

**Affiliations:** a Department of Chemistry, ARSD College, University of Delhi, Delhi, India; b Department of Chemistry, University of Delhi, Delhi, India; c Department of Chemistry, Indian Institute of Technology, Bhubneshwar, Argul, Odisha, India; d Department of Chemistry, Jamia Millia Islamia, Delhi, India; e Department of Zoology, DDU College, Universiy of Delhi, Delhi, India; f CRIBS, Jamia Millia Islamia, Delhi, India

**Keywords:** Theoretical chemistry, ZINC database, Thiazolidines, Simulation, Imidazole, Docking, Density functional theory, Dengue virus

## Abstract

A theoretical model was developed to allosterically inhibit the biological activity of dengue virus (DENV) by targeting the non-structural protein ns2B-nsP3 protease based on the *in silico* studies. The imidazole, oxazole, triazole, thiadiazole, and thiazolidine based scaffolds were imported from the ZINC database, reported by various research group with different biological activity. They were found biologically active as they contain heterocyclic fragments. Generic evolutionary based molecular docking was performed to screen the highly potent molecule. The docking results show that the molecule having ZINC ID-633972 is best inhibitor. Further, the bioavailability and other physiochemical parameters were also calculated for the top four molecule. The highly potent molecule was further refined by the density functional theory and molecular dynamic (MD) simulation. The MD analysis coroborate the successful docking of the molecule in the binding cavity of nsP2B-nsP3 protease of DENV. The Molecular Mechanics Poisson-Boltzmann Surface Area approach was also applied and result coroborate the docking and MD result.

## 1 Introduction

Dengue virus (DENV) is the major health concern in the world especially in the tropical and subtropical areas. It's caused by the flavivirus and rapidly spread by the mosquito especially *Aedes aegypti* [1]. Four different stereotypes of the virus are DENV-1, DENV-2, DENV-3, and DENV-4. The genomic structure of all four serotypes are about 65 % similar. As per the World Health Organization (WHO), about 96 million clinical cases of dengue infections reported world wide annually. Due to the lack of specific treatment and vector control, dengue fever becomes fatal [2, 3]. After understanding the severity of dengue, the WHO recommended CYD-TDV and the licensed was given as Dengvaxia^TM^. However, it is found that the efficacy of vaccine varies by serotype and the higher efficacy rates were reported against DENV-3 and DENV-4 in comparison to DENV-1 and DENV-2 [[4], [5]]. The non-structural protein, nsP2B-nsP3 protease of Dengue is involved in the catalytic infectious activity [6]. For the prevention of dengue fever, appropriate management of symptoms is needed by maintaining an adequate volume of fluids. There were some commercially available vaccine against dengue i.e., NITD-008 and Balapiravir and they were withdrawn due to toxicity and lack of potency against all four serotype. Celgosivir is a host alpha-glucosidase inhibitor and under clinical trial [7].

In present time, there is no drug available to protect against all four dengue serotypes. Although researchers are trying to develop a potential and cheap vaccine [8]. From the literature survey, it was found that thiazolidine and oxazole derived drugs have various biological activities like-anti-diabetic, anti-cancer, anti-HIV, anti-oxidant, etc [9, 10]. Imidazoles and thiadiazoles are important classes of heterocycle molecules [11, 12, 13, 14, 15, 16, 17, 18]. Imidazole ring is present in many natural biological building blocks and biological potent molecules. Triazoles have a wide range of industrial applications such as corrosion inhibitors, agrochemicals, etc [19].

Herein, reported molecules based on imidazole, oxazole, triazole, thiadiazole, and thiazolidine scaffolds are used to find a biological potent inhibitors against nsP2B-nsP3 protease of dengue virus. A pipeline of work based on the computational technique like molecular docking, absorption, distribution, metabolism, and excretion (ADME), density functional theory (DFT), molecular dynamic (MD) simulation, and Molecular Mechanics Poisson-Boltzmann Surface Area (MM-PBSA) is followed to find potential inhibitors for nsP2B-nsP3 protease of DENV.

## 2 Experimental

**Fig. 1:**
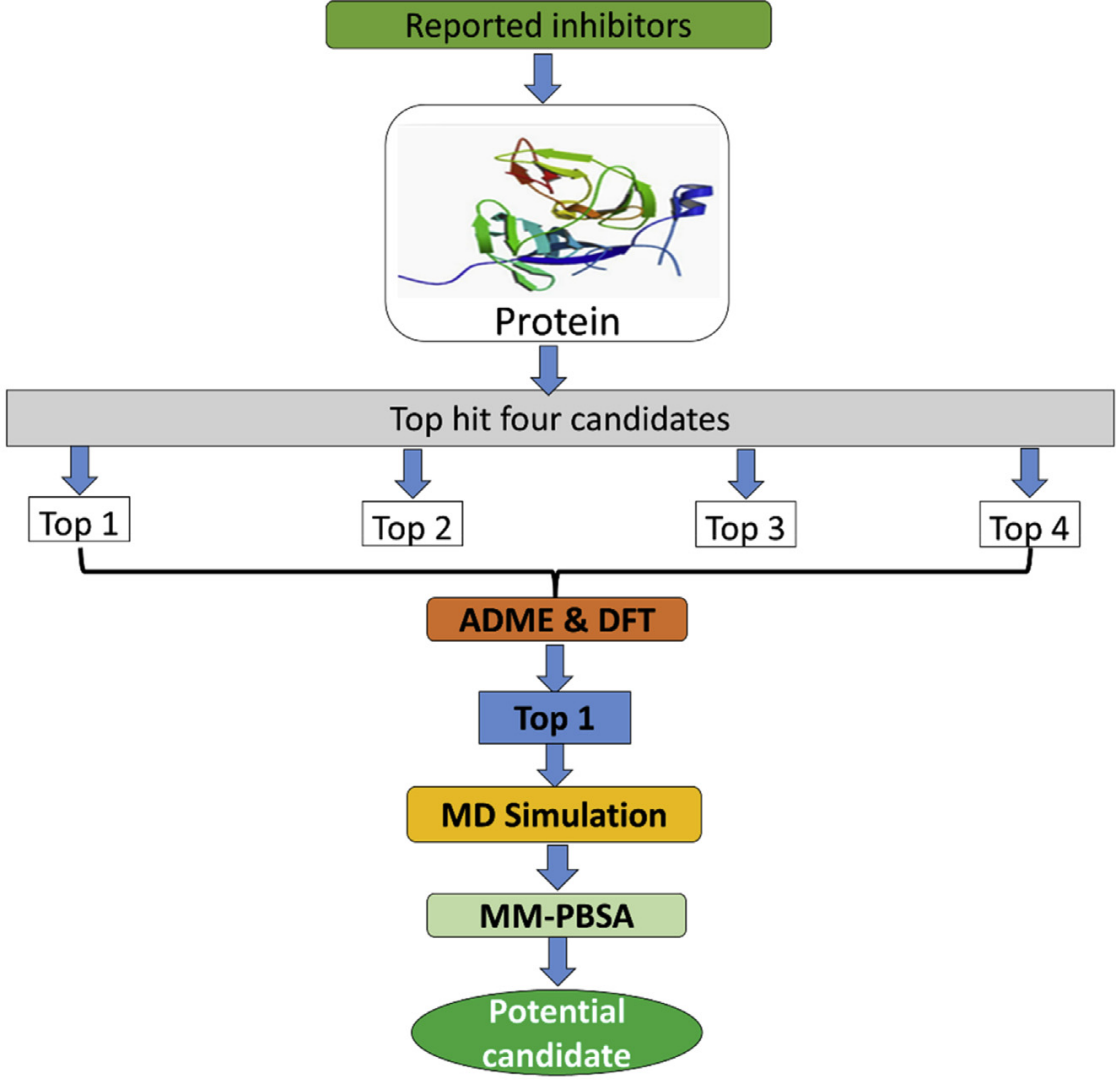
The schematic representation of the experimental methodology.

A methodology was developed to find the potential inhibitor from the library of molecules, and it can be understood from Fig. 1. Based on the approach given, a total of 138 molecules based on imidazole, oxazole, triazole thiadiazole, and thiazolidine based scaffolds were screened against the ns2PB-nsP3 protease of DENV.

### 2.1 Preparation of ligands and receptor

A diverse set of a total of 138 ligands were taken from the ZINC database of imidazole, oxazole, triazole thiadiazole, and thiazolidine based scaffolds (Table 1) [20]. All molecules were subjected to the geometry optimization by performing the energy minimization to eliminate the stearic conflict in bond and angle of the crystal structure using the molecular mechanics as a force field, the root mean square (RMS) gradient norms for the optimization was set to 0.01. The Chem3D 12.0 of Perkin Elmer was used to optimize the ligand molecule. The nsP2B-nsP3 protease of DENV (**PDB-ID 4M9F)** was imported from protein data bank (RCSB) [21]. The preparation of receptor was performed using UCSF Chimera 1.11.2 in dock prep module, where the removal of solvents, adding hydrogen, replacing incomplete residues using Dunbrack rotamer library, conversion of selenomethionine to methionine, conversion of bromo-UMP to UMP, conversion of methylselenyl-dCMP to CPM and charges were assigned according to the AMBER.ff14SB force field [22]. These optimized ligands and prepared receptor were used for further studies.

**Table 1:** List of all molecules with their ZINC ID.

C.N.	ZINC ID	C.N.	ZINC ID	C.N.	ZINC ID	C.N.	ZINC ID	C.N.	ZINC ID	C.N.	ZINC ID
1	633936	24	634006	47	8442171	70	8442238	93	8442266	116	40438746
2	633938	25	634008	48	8442180	71	8442240	94	8442267	117	41705868
3	633944	26	634012	49	8442182	72	8442241	95	8442273	118	41707416
4	633946	27	667005	50	8442184	73	8442243	96	8442275	119	52918773
5	633947	28	1240782	51	8442185	74	8442244	97	8442276	120	54712612
6	633951	29	1383195	52	8442189	75	8442246	98	8442279	121	54712613
7	633953	30	1383196	53	8442194	76	8442247	99	8442281	122	55107172
8	633955	31	1383199	54	8442196	77	8442248	100	8442294	123	55107174
9	633958	32	1383200	55	8442198	78	8442249	101	8575396	124	55112536
10	633962	33	1386512	56	8442200	79	8442250	102	11804308	125	58567596
11	633964	34	1386519	57	8442201	80	8442251	103	12378847	126	61488581
12	633966	35	2124713	58	8442204	81	8442252	104	19318821	127	61488582
13	633968	36	4168932	59	8442217	82	8442253	105	19794473	128	62542246
14	633970	37	4168934	60	8442218	83	8442254	106	24990471	129	63011626
15	633972	38	5286115	61	8442220	84	8442255	107	26144438	130	69700429
16	633975	39	8442149	62	8442221	85	8442257	108	36635229	131	69700431
17	633978	40	8442152	63	8442222	86	8442259	109	36635230	132	69775530
18	633982	41	8442157	64	8442224	87	8442260	110	36964805	133	69950989
19	633984	42	8442159	65	8442225	88	8442261	111	37114532	134	69978692
20	633992	43	8442162	66	8442228	89	8442262	112	37287049	135	71540581
21	633997	44	8442164	67	8442229	90	8442263	113	37328374	136	71540582
22	633999	45	8442165	68	8442230	91	8442264	114	40438714	137	71541665
23	634001	46	8442166	69	8442235	92	8442265	115	40438744	138	71541856

### 2.2 Docking protocol

Molecular docking between the optimized ligand and prepared ns2PB-nsP3 protease of DENV was carried out by the iGEMDOCK v2.1 [23]. The allosteric inhibition of the receptor was performed. Herein, the binding site was not defined and the whole protein was considered for the search of best cavity. iGEMDOCK uses generic algorithms (GA), the GA parameter was set to the default in drug screening mode, in which the population size is set to 200, generation is set to 70 and number of solution is set to 3.

### 2.3 Post dock screening and analysis

The post dock screening of the docked poses of all the molecule was done by the iGEMDOCK, where the top scored compound was screened on the basis of the total minimum binding energy of docking. The total binding energy of the protein-ligand complex can be given by Eq. (1).

(1) $${\text{E}}_{Binding}=E_{VDW}\;+\;H_{bond}+\;E_{Elec}$$

where E_VDW_ stands for van der Waal energy, H_bond_ stands for hydrogen bonding energy and E_Elec_ stands for electro statistic energy [23].

The docked pose analysis of hit molecules were also performed by the Discovery Studio visualizer v17.2.016349 of Biovia [24]. Van der Waals interaction, electrostatic interaction and covalent interaction were analyzed in 2D, while the H-bonding with distance and H-bonding surface analysis were done in 3D. The binding cavity analysis and their corresponding amino acid contribution in the stabilization of the cavity were performed with the help of iGEMDOCK.

### 2.4 ADME properties

Lipinski et al. (1997) and Lipinski (2004) studied more than 2000 drug molecule and basis on their studies, they proposed the Lipinski's rule of five or Pfizer rule of five [25]. This rule is very useful in the prediction of a new compound to act as a drug molecule based on the membrane permeability and absorbance in the organisms. Authors have already considered the reported drug molecules, although the drug-likeness parameters and other biological properties for top four drug molecule were calculated by using online server http://molinspiration.com/ and http://swissadme.ch/ [26, 27].

### 2.5 DFT studies

All DFT calculations for top four molecules were carried out using the GAUSSIAN 09 package [28]. Becke's 3 parameters functional Lee, Yang, Parr B3LYP and basis set 6-31+G(d) was used to optimize the geometries of the studied molecules [29]. Further, energy of highest occupied molecular orbital (HOMO) and lowest unoccupied molecular orbital (LUMO) of optimized structures were taken to calculate all the physiochemical descriptors. DFT is a very useful technique to provide the chemical descriptors like chemical potential (μ), electronegativity (χ), hardness (η), softness (S), and global electrophilicity index (ω). For the N-electron system having energy level E, the values of all the physiochemical descriptors can be given by Eqs. (2), (3), (4), (5), and (6) [30].

(2) $$\mu ={\left(\frac{\delta \;E}{\delta \;N}\right)}_{v}=\frac{1}{2}\;\left(E_{LUMO}+\;E_{HOMO}\right)=\;-\frac{1}{2}\;\left(IE+EA\right)$$

(3) $$\chi =-\mu ={\left(\frac{\delta \;E}{\delta \;N}\right)}_{v}=-\frac{1}{2}\;\left(E_{LUMO}+\;E_{HOMO}\right)=\;\frac{1}{2}\;\left(IE+EA\right)$$

(4) $$\eta =\frac{1}{2}\left(\frac{\delta^{2}\;E}{\delta \;N^{2}}\right)=\frac{1}{2}\;\left(E_{LUMO}-\;E_{HOMO}\right)=\;\frac{1}{2}\;\left(IE-EA\right)$$

(5) $$S=\frac{1}{2\eta }$$

(6) $$\omega =\left(\frac{\mu^{2}}{2\eta }\right)$$

### 2.6 MD simulation analysis

Molecular dynamic simulation was performed by GROMACS 5.1.4 [31]. Minimization of structural conflict in the structure of the protein was performed by applying the CHARMM force-field [32]. Solvation of the protein was done in the cubical fashion by the water taking as a simple point charge model. The neutralization of the solvated system was performed by replacing water with Na^+^ and Cl^−^ ions. Further, the whole system was minimized to release the stearic conflict contacts by steepest decent algorithm for 50000 steps.

The isothermal-isochoric (canonical) equilibration of the system was also performed by using leap-frog integrator and cut off scheme was set to verlet. The system was equilibrated for 100 ps in periodic boundary condition and the temperature was set to 300 K. Pressure of the system was set to 1 bar, the Parrinello-Rahman coupling was applied for pressure and the system is equilibrated for 100 ps in the periodic boundary condition. Finally, the Molecular Dynamics was performed for 30 ns with a leap-frog integrator and the coordinates of the system were recorded at every 10 ps for the further analysis of the system.

### 2.7 MM-PBSA analysis

The MM-PBSA analysis is a post-processing analysis, which is performed on the final trajectories obtained from the MD simulation of the protein-ligand complex. The MM-PBSA analysis of protein-ligand complex was done by the g_mmpbsa tool [33, 34, 35]. It's a most efficient and accurate technique to analyze the protein and ligand interaction as it uses poison-boltzmann equation. The binding free energy change, molecular mechanics potential energy change, solvation free energy change in term of polar and non-polar, solvent accessible surface area (SASA), solvent accessible volume (SAV) and Weeks–Chandler–Andersen (WCA) energies were calculated for the protein-ligand complex according to Eqs. (7), (8), (9), (10), (11), (12), (13), and (14) respectively.

(7) ΔG_binding_ = G_complex_ – (G_protein_ + G_ligand_)

(8) E_MM_ = E_bonded_ + E_nonbonded_ = E_bonded_ + (E_vdW_ + E_elec_)

(9) G_solvation_ = G_polar_ + G_nonpolar_,

(10) ▽.[ε(r)▽.φ(r)] – ε(r)κ(r)^2^sinh[φ(r)] + 4πρ^f^(r)/kT = 0

(11) G_nonpolar_ = G_cavity_ + G_vdW_

(12) G_nonpolar_ = γ*A* + *b*

(13) G_nonpolar_ = *pV* + *b*

(14) G_nonpolar_ = γ*A* + *pV +* G_vdW_

Where *E*_MM_ is vacuum potential energy, *E*_elec_ is electrostatic, *E*_vdW_ is van der Waals, ϕ(*r*) is electrostatic potential, ε(*r*) is the dielectric constant, ρ^f^(*r*) is the fixed charge density, k is Boltzmann constant, γ is a coefficient related to the surface tension of the solvent, *A* is SASA, *b* is a fitting parameter, *p* is a coefficient related to pressure of the solvent and *V* is SAV.

## 3 Result and discussion

### 3.1 Docking results

Docking is the computational approach to find the suitable binding cavity in the protein and the small molecule can fit suitably with less perturbing the dynamics of the protein. Docking may be binding site-specific or random to search a new active cavity [36, 37, 38, 39, 40, 41, 42, 43, 44, 45, 46, 47, 48]. Researcher reported the Flavonoids as non-competitive inhibitors for nsP2B-nsP3 protease of DENV, and mentioned formation of hydrogen bond between Gln88, Gln167, and Gly124 form Hydrogen bonding with the flavonoids [49]. https://www.ncbi.nlm.nih.gov/pubmed/?term=Wu%20H%5BAuthor%5D&cauthor=true&cauthor_uid=25487800 Qamar et al. 2016 reported the inhibition of dengue ns2PB-nsP3 protease of DENV by some heterocyclic molecules and reported active site as His51, Asp75, Ser135, Gly153, Gly151, Pro132, Val154 and Leu128 [50]. Herein, the allosteric binding cavity search approach was used to find potent candidate as well the new allosteric cavity within the receptor. Docking result in term of energy of all 138 molecules is mentioned in Table 2. The molecule 633972, 633992, 8442220 and 8442281 are the top four hit molecules and their total binding energy contribution are -141.595 kJ/mol, -140.435 kJ/mol, -140.077 kJ/mol and -139.323 kJ/mol respectively.

**Table 2:** Docking energies of the all the ligands.

Ligand	Energy	Ligand	Energy	Ligand	Energy	Ligand	Energy	Ligand	Energy	Ligand	Energy
633936	-119.4	634006	-125.2	8442171	-112.0	8442238	-112.3	8442266	-124.2	40438746	-91.3
633938	-138.9	634008	-122.1	8442180	-129.5	8442240	-108.6	8442267	-111.2	41705868	-81.1
633944	-116.5	634012	-119.7	8442182	-116.9	8442241	-104.1	8442273	-137.2	41707416	-84.1
633946	-136.9	667005	-108.2	8442184	-120.8	8442243	-124.3	8442275	-121.9	52918773	-88.9
633947	-117.7	1240782	-96.33	8442185	-120.0	8442244	-121.4	8442276	-107.5	54712612	-89.8
633951	-105.7	1383195	-76.88	8442189	-118.4	8442246	-118.3	8442279	-115.2	54712613	-87.7
633953	-120.0	1383196	-77.0	8442194	-122.6	8442247	-120.4	8442281	-139.3	55107172	-76.0
633955	-105.0	1383199	-77.4	8442196	-115.8	8442248	-113.1	8442294	-116.6	55107174	-71.9
633958	-121.1	1383200	-73.4	8442198	-124.6	8442249	-105.9	8575396	-114.7	55112536	-74.8
633962	-125.7	1386512	-83.2	8442200	-116.7	8442250	-110.1	1.2E+07	-63.8	58567596	-97.2
633964	-120.6	1386519	-79.6	8442201	-124.6	8442251	-117.1	1.2E+07	-98.2	61488581	-90.1
633966	-108.5	2124713	-111.6	8442204	-113.8	8442252	-118.03	1.9E+07	-101.3	61488582	-75.5
633968	-117.2	4168932	-94.6	8442217	-102.2	8442253	-113.2	2E+07	-101.0	62542246	-72.8
633970	-112.4	4168934	-90.1	8442218	-115.1	8442254	-110.8	2.5E+07	-98.3	63011626	-99.2
633972	-141.5	5286115	-106.4	8442220	-140.0	8442255	-110.4	2.6E+07	-97.7	69700429	-97.9
633975	-135.1	8442149	-130.1	8442221	-100.1	8442257	-116.3	3.7E+07	-82.2	69700431	-101.2
633978	-126.3	8442152	-107.8	8442222	-108.6	8442259	-125.0	3.7E+07	-90.6	69775530	-96.0
633982	-125.5	8442157	-111.5	8442224	-98.9	8442260	-115.5	3.7E+07	-73.0	69950989	-120.9
633984	-127.3	8442159	-112.0	8442225	-109.7	8442261	-112.8	3.7E+07	-84.2	69978692	-109.2
633992	-140.4	8442162	-130.2	8442228	-104.3	8442262	-116.1	3.7E+07	-82.8	71540581	-107.8
633997	-119.0	8442164	-136.3	8442229	-109.0	8442263	-107.0	3.7E+07	-93.2	71540582	-91.1
633999	-131.8	8442165	-106.8	8442230	-118.2	8442264	-113.1	4E+07	-100.8	71541665	-83.1
634001	-132.8	8442166	-99.9	8442235	-108.5	8442265	-130.9	4E+07	-98.4	71541856	-88.2

**Fig. 2:**
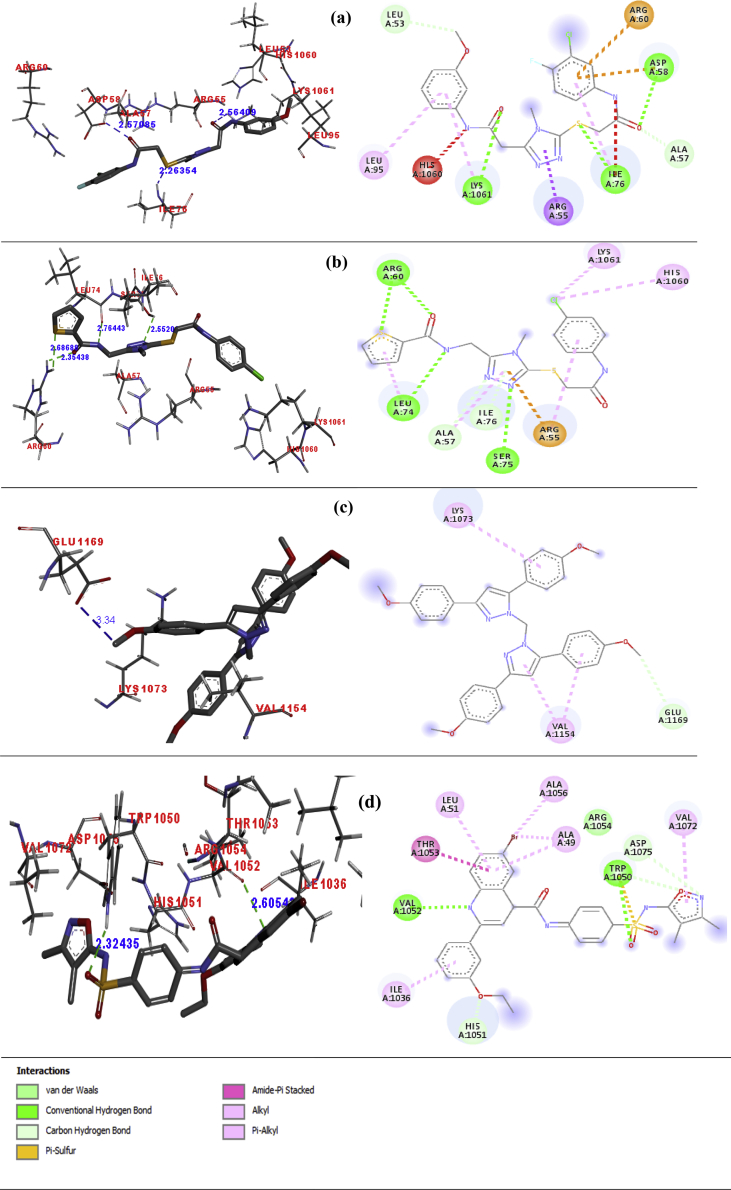
a–d Docked poses of compound 633972, 633992, 8442220 and 8442281 respectively in 3D and 2D. Amino Acid name is labelled red while H-bond distance in blue color.

The docked poses of the top four molecules (ZINC ID 633972, 633992, 8442220 and 8442281) is given in Fig. 2. From the docked pose, it's clear that the top-ranked molecule (ZINC ID 633972) shows H-bonding interaction with LYS1061, ILE76 and ASP58 with distance of 2.56409 Å, 2.2635 Å and 2.57085 Å respectively. Besides this, there are four hydrophobic interaction ILE76, ARG55, LEU95 and LYS1061 with distance of 5.00 Å, 2.85 Å, 5.07 Å and 5.33 Å respectively. Second top (ZINC ID 633992) shows H-bonding interaction two with ARG60, one with ARG55 and one with LEU74 with distance of 2.68688 Å, 2.35438 Å, 2.86443 Å and 2.5520 Å. Beside this, it shows hydrophobic interactions with LYS1061 (3.89 Å), HIS1060 (4.49 Å), ARG55 (5.19 Å), ALA57 (5.19 Å), ILE76 (4.87 Å), and LEU74 (4.08 Å). The third-ranked molecule (ZINC ID 8442220) show non-classical H-bond interaction with GLU1169 (3.34 Å) and hydrophobic interaction with LYS1073 (5.12 Å) and VAL (4.95 Å, 3.84 Å). While, the fourth-ranked molecule (ZINC ID 8442281) shows total of two H-bonding interaction with TRP1050 and VAL1052 with distance of 2.324 Å and 2.605 Å and hydrophobic interaction with ILE1036 (4.91 Å), ALA1056 (4.10 Å), ALA49 (5.47 Å, 3.50 Å), LEU51 (5.39 Å), ARG1054 (4.65 Å) and VAL1072 (4.91 Å) respectively as in Table 3.

**Table 3:** The interaction with a distance of the top four molecule.

Ligand	H-Bond		Electrostatic		Hydrophobic		Miscellaneous
	Amino Acid	Distance	Amino Acid	Distance	Amino Acid	Distance	
633972	LYS1061	2.56	ASP58	3.72	ILE76	5.00	Acceptor/donor clash at HIS1060 (2.40) and ILE76 (2.90)
	ILE76	2.26	ARG60	4.76	ARG55	2.85	
	ASP58	2.57			LEU95	5.07	
					LYS1061	5.33	
633992	ARG60	2.68, 2.35	ARG55	3.67	LYS1061	3.89	Sulphur interaction at ARG60 (3.25) and ARG55 (3.67)
	LEU74	2.76			HIS1060	4.49	
	SER85	2.55			ARG55	5.19	
					ALA57	5.19	
					ILE76	4.87	
					LEU74	4.08	
8442220	GLU1169	3.34	-	-	LYS1073	5.12	
					VAL1154	4.95,3.84	
8442281	TRP1050	2.32			ILE1036	4.91	Sulfur interaction at TRP1050 (4.78) and stearic bump in the molecule
	VAL1052	2.60			ALA1056	4.10	
					ALA49	5.47, 3.50	
					LEU51	5.39	
					ARG1054	4.65	
					VAL1072	4.91	

**Fig. 3:**
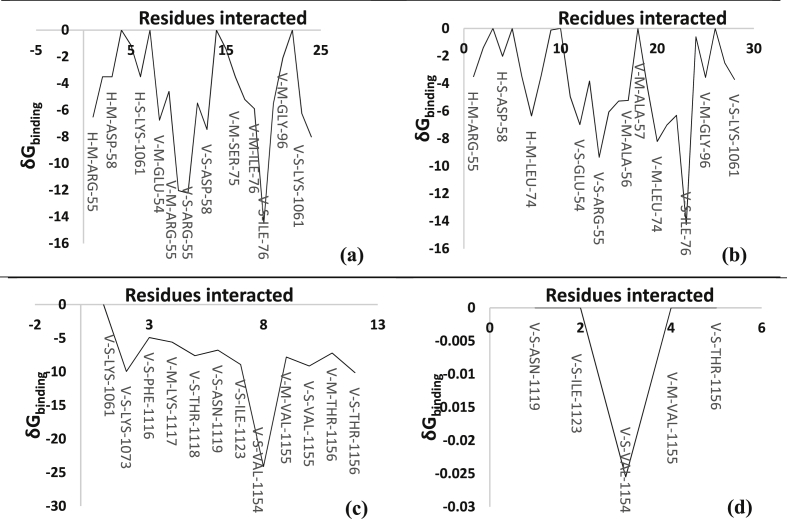
a–d represents the binding cavity amino acid residues contribution of compound 633972, 633992, 8442220 and 8442281 respectively.

The binding cavity residues contribution for the top four molecules is analyzed. The δG_binding_ versus contributing energy of amino acid residues of the binding cavity were also plotted and are given in Fig. 3a-d. The major cavity contributing amino acid residues for 633972 is with ARG-55, ASP-58, LYS-1061, GLU-54, SER-75, ILE-76 and GLY-96, for 633992 are ARG-55, ASP-58, LEU-74, ALA-56, LYS-1061, GLU-54, SER-75, ILE-76 and GLY-96, for 8442220 are LYS-1061, LYS-1073, PHE-1116, LYS-1117, THR-111-8, ASN-1119, ILE-1123, VAL-1154, VAL-1155 and THR-1156 and for 8442281 are ASN-1119, ILE-1123, VAL-1154, VAL-1155 and THR-1156. Binding cavity analysis shows that 633972 and 633992 targeted the same cavity which is the active cavity of NSP2B-NSP3 protease of DENV.

**Fig. 4:**
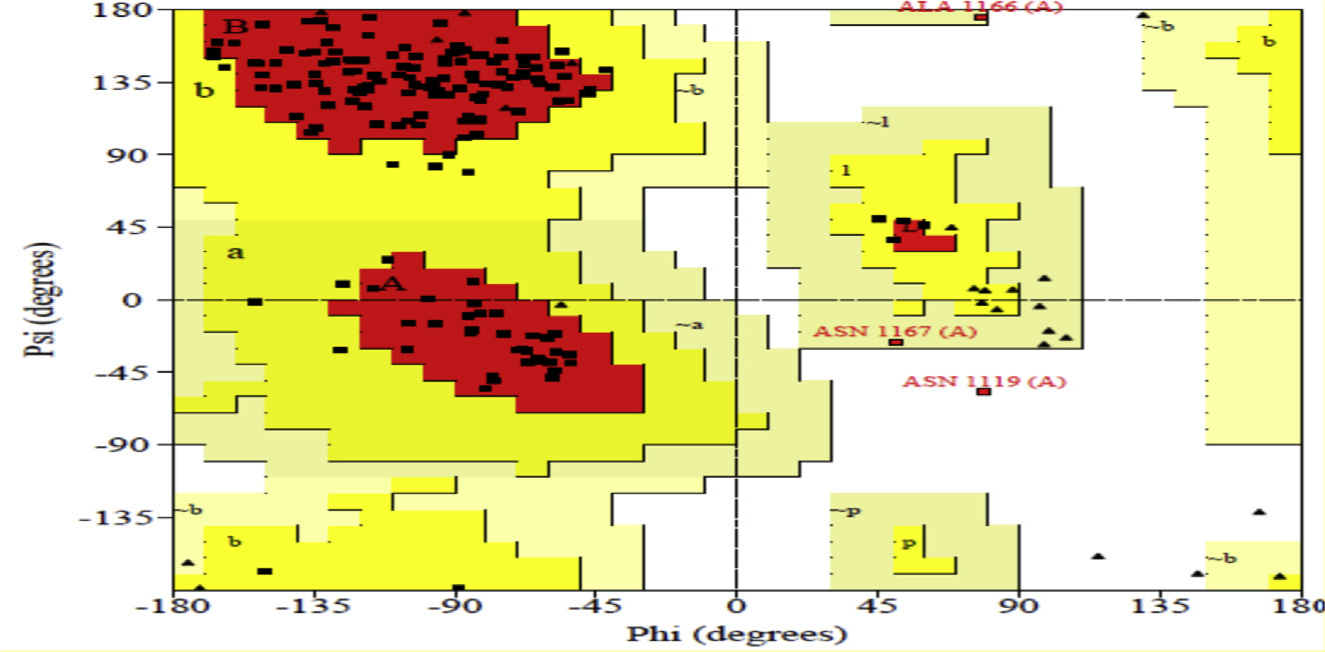
Ramachandran plot of nsP2B-nsP3 protease of DENV (4M9F).

The structural properties of the nsP2B-nsP3 protease of DENV was analysed using the SAVES server (online) for Ramachandran plot [51]. The analysis is explained in Fig. 4 and Table 4 regarding the allowed and disallowed regions.

**Table 4:** Analysis of Ramachandran plot of nsP2B-nsP3 protease of DENV.

Item	nsP2B-nsP3 protease 4M9F	
	Number of amino-acids	% of amino-acids
Residues in most favored regions	144	88.9
Residues in additional allowed regions	15	9.3
Residues in generously allowed regions	2	1.2
Residues in disallowed regions	1	0.6
Number of non-glycine and non-proline residues	162	100
Number of end residues excluding glycine and proline	4	
Number of glycine residues (shown as triangles)	27	
Number of proline residue	7	
Total number of residues	200	

### 3.2 ADME results

Lipophilicity is one of the most important parameters to consider a molecule to be a drug, it is the ratio of the partition coefficient of octanol/water and its value should be less than five [25]. H-Bond donor (HBD) and H-Bond acceptor (HBA) are also valuable parameters for a drug, the value of HBD should be less than five and the value of HBA should be less than ten [52, 53]. Topological polar surface area (TPSA) for drug-likeness is also an important parameter and the value of TPSA should be less than 130 Å^2^, the solubility of the molecule log S, should not be more than 6 [54]. The results are incorporated in Table 5.

**Table 5:** Physiochemical properties of the top four molecules against ns2PB-nsP3 protease of DENV.

Properties	The top hit four molecules			
	633972	633992	8442220	8442281
*Log ​S*	-4.15	-3.95	-7.66	-7.24
*Solubility*	Moderately soluble	Soluble	Poorly soluble	Poorly soluble
*Heavy atoms*	31	27	43	40
*No. of rotational bonds*	10	9	10	9
*No. H-bond acceptors*	6	4	6	7
*Num. H-bond donors*	2	2	0	2
*Log ​P*_*o/w*_	2.44	2.78	4.62	3.88

TPSA is considered to be a useful parameter for the guess of drug transport properties of the ligands and obtained results are incorporated in Table 6. Total polar surface area (TPSA) of the screened molecules was determined using the online server, molinspiration (www.molinspiration.com) and the absorbance are calculated according to Eq. (15) [55].

(15) %ABS = 109 – [0.345 × topological polar surface area (TPSA)]

**Table 6:** Biological properties of the top hit four molecules against DENV.

Properties	633972	633972	8442220	8442281
*TPSA (Å*^*2*^*)*	123.44	142.45	72.56	131.80
*%ABS*	66.4132	59.85475	83.9668	63.529
*GI absorption*	High	Low	Low	Low
*BBB permeant*	No	No	No	No
*P-gp substrate*	No	No	No	No
*CYP3A4 inhibitor*	Yes	Yes	Yes	Yes
*miLog P*	4.38	2.46	7.37	6.12
*GPCR ligand*	-0.09	-0.71	-0.19	-0.09
*Ion channel modulator*	-0.38	-1.13	-0.62	-0.78
*Kinase inhibitor*	-0.49	-0.76	-0.32	-0.27
*Nuclear receptor ligand*	-0.52	-1.21	-0.32	-0.57
*Protease inhibitor*	-0.40	-0.67	-0.23	-0.46
*Enzyme inhibitor*	-0.24	-0.63	-0.36	-0.39

The top four molecules shows good activity for Gastrointestinal (GI) absorption, Blood Brain Barrier (BBB), permeability glycoprotein (P-gp), Cytochrome P450 (CYP) isoenzyme, Molinspiration based Log P (miLog P), GPCR ligand, Ion channel modulator, Kinase inhibitor, Nuclear receptor ligand, Protease inhibitor and Enzyme inhibitor. TPSA of the top compounds are found within the range and % ABS of 633972 is good. The GI absorption of 633972 is high. The miLog P value of 633972 and 633992 is less than five while for the 8442220 and 8442281 is more than five. In general, compound having positive values are considerably biological active, the value in between the 0 to -0.50 is moderately active while the value less than -0.50 is presumed as inactive. The GPCR ligand value of **633972, 8442220** and **8442281** shows the moderate activity while compound **633992** is the inactive. The ion channel modulator value shows that compound **633972** is moderately active while the rest are biologically inactive. The kinase inhibitor value for **633972** is inactive while for the rest all it is moderately active. The nuclear receptor ligand value for the molecule **8442220** is moderately active while for rest all are inactive. The protease inhibitor and enzyme inhibitor value for the molecule **633992** is inactive while for the rest all are moderately active.

### 3.3 DFT results

DFT approach is used to study the various electronic parameters of a molecule to refine it as a potent among the numerous one [56]. The values of energy of highest occupied molecular orbital (HOMO) and lowest unoccupied molecular orbital (LUMO) for a biologically potent molecule and the energy gap between the HOMO and LUMO are useful to understand their biological and chemical potency [57]. Value of HOMO, LUMO and various electronic descriptors of singlet state of top four molecules are given in Table 7.

**Table 7:** Value of HOMO, LUMO and chemical descriptors of top four molecules.

Energy & Descriptor	633972 (Singlet state)	633992 (Singlet state)	8442220 (Singlet state)	8442281 (Singlet state)
*LUMO*	-0.0466	-0.06689	-0.0601	-0.10837
*HOMO*	-0.25623	-0.25213	-0.09229	-0.22824
*L-H*	0.20963	0.18524	0.03219	0.11987
*L + H*	-0.30283	-0.31902	-0.15239	-0.33661
*ɳ*	0.104815	0.09262	0.016095	0.059935
*Χ*	0.151415	0.15951	0.076195	0.168305
*S*	4.77031	5.398402	31.06555	8.342371
*μ*	-0.15142	-0.15951	-0.0762	-0.16831
*Ω*	0.109367	0.137354	0.180357	0.236311

**Fig. 5:**
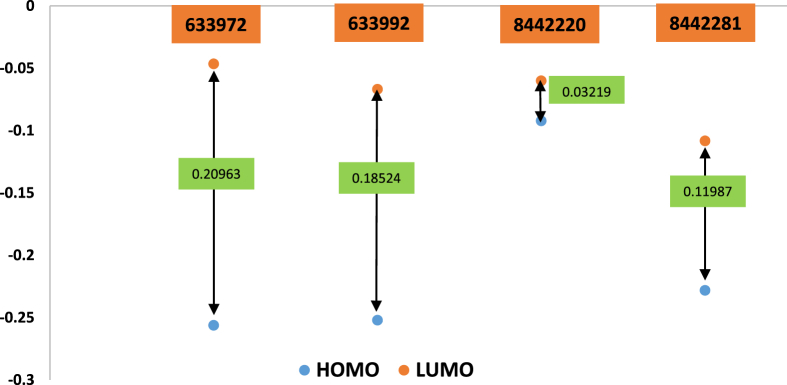
The graphical representation of HOMO and LUMO gap of the top four molecule.

The HOMO∼LUMO gap for molecule 633972 is largest among the top four molecules. The HOMO of molecule 633972 has the lowest value among all top four molecule and indicate more tendency to donate electron to the LUMO of an amino acid of the active cavity of receptor. The chemical hardness and softness parameter are useful to predict the molecular reactivity and stability of the molecule in the various environment. More the softness or less the hardness value make the molecule more polarizable. The chemical hardness value of 633972 molecules is maximum and it reveals that this is enough hard in the active cavity of receptor. The overall hardness order of the molecules is 633972 > 633992>8442281 > 8442220. When the molecule achieves equilibrium in any medium the ability of a molecule to leave the electron is known as chemical potential (μ), and the chemical potential order of the molecule is 8442220 > 633972>633992 > 8442281. The ability of attraction of electron of shared covalent bond in the molecule is known as the electronegativity. The overall electronegativity order of the molecules is 8442220 > 633992>633972 > 8442281. The electrophilicity value defines the ability to accept the electron of the inhibitor molecule in the binding cavity of protein. The order of electrophilicity value is 8442281 > 8442220>633992 > 633972. The overall electronic descriptors of all molecules shows good agreement with the docking result, hence the molecule 633972 can be chosen for further studies. The HOMO-LUMO gap of the top four molecules are given in Fig. 5.

**Fig. 6:**
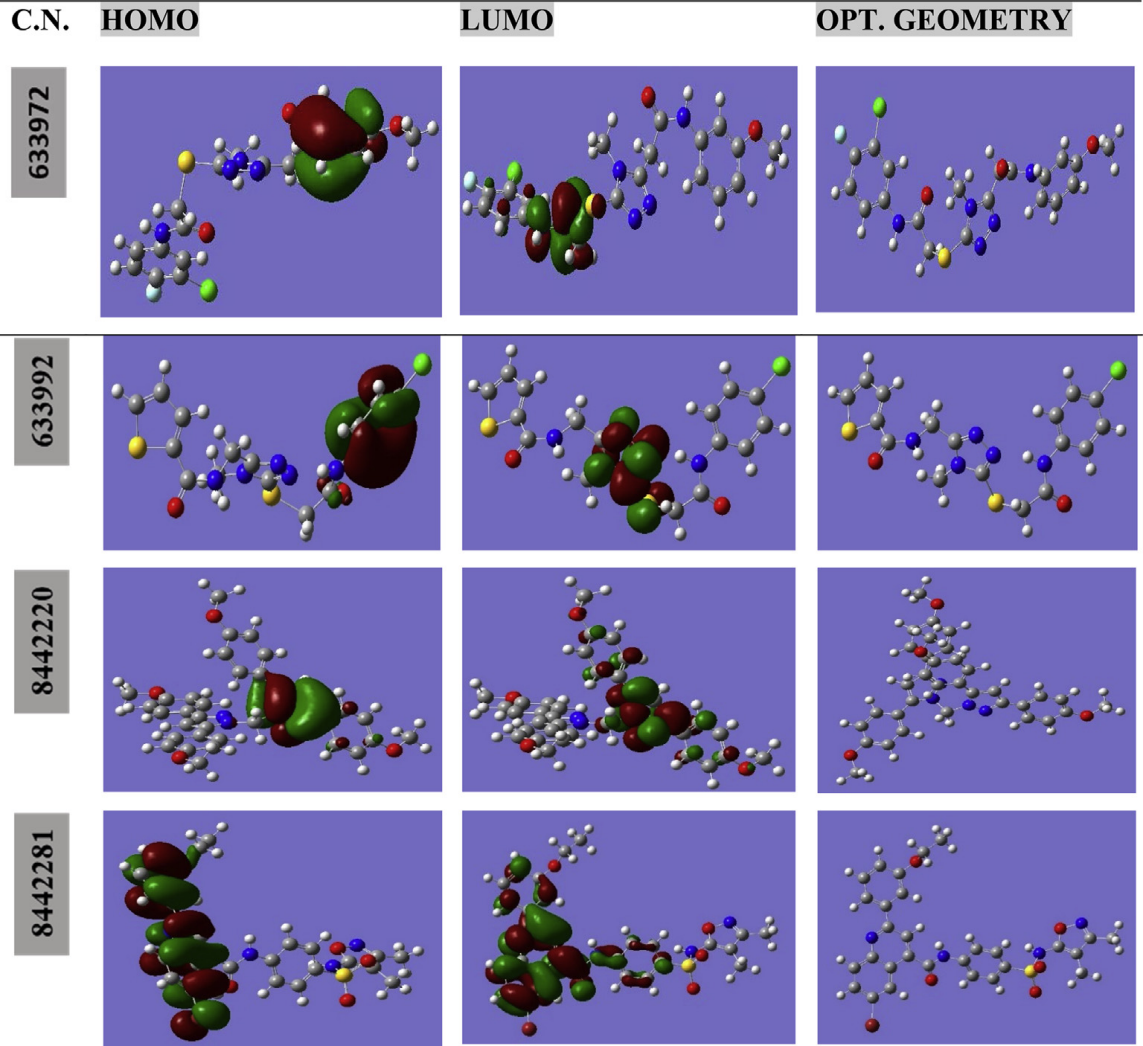
The HOMO, LUMO and optimized geometry of the top four compounds.

The frontier orbital pictorial representation of the top four molecules was also analyzed to find the electronic distribution over the molecule. The orbitals are dually colored in red and green color, the red color represents the positive lobe while the green color represents the negative lobe. The HOMO, LUMO and optimized geometry of molecule 633972, 633992, 8442220 and 8442281 are given in Fig. 6. For 633972, the HOMO is concentrated on benzene ring while the LUMO on the amide part of the molecule. For 633992, the HOMO is centred on benzene ring while LUMO on the triazole ring. In case of 8442220 the HOMO and LUMO both are centered on one pyrazolidine ring of the molecule. For 8442281, the HOMO and LUMO is almost centered on the benzene ring.

### 3.4 MD simulation

**Fig. 7:**
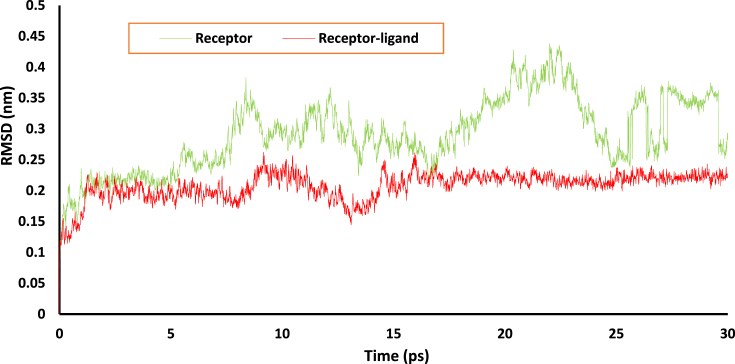
RMSD of ns2PB-nsP3 protease of DENV with and without 633972.

MD study the biological function based on the molecular interactions. The conformations of receptor are the key to these biological functions. These conformational changes can be checked or regulated in a certain direction with the help of external ligand (allosteric regulators) [58]. The ligand can bind into the binding pocket and inhibit some unusual function of the receptor. The MD simulation of a few nanoseconds can predict some of the structural or conformational changes of receptor in the presence of ligand [59]. Top-ranked molecule from the library, 633972 is selected for the MD simulation. Root mean square deviation (RMSD) measures the deviation of atomic positions of protein Cα atoms. The RMSD of Cα atoms of the protein backbone can be used as a quantitative measurement tool to find the structural deviation of two superimposed protein macromolecule [60]. The RMSD of nsP2B-nsP3 protease of DENV with and without 633972 are given in Fig. 7**.** The RMSD of receptor shows more deviation in the atomic positions. The RMSD value of protein is ranged around 0.3 nm, while the RMSD value of receptor-ligand complex is almost invariant around 0.2 nm. The more deviation in the case of receptor indicates the loop structure in protein macromolecule. The RMSD result clearly shows the successful stabilization of protein after the binding of a ligand in the active cavity of receptor.

### 3.5 MM-PBSA

In the docking, the docking algorithms are used to calculate the binding energy of the ligand to the receptor, but these algorithms are not accurate. To find the more accurate binding energy, the molecular mechanics combined with the Poisson–Boltzmann equation is used. It uses a time scaling parameter to calculate it. The values of all energies calculated in the mmpbsa analysis are given in Table 8. The binding energy value of ligand to the protein for 633972 was found to be -56.947 kJ/mol, and the van der Waal energy was found to -110.221 kJ/mol. All values corroborate the docking and MD results. Electrostatic energy was found to -20.364 kJ/mol, while the electrostatic interaction is zero in the docking result, this result shows that the more accurate prediction of energy values by the mmpbsa analysis. The polar solvation energy, solvent assessable surface area energy, solvent accessible volume (SAV) and Weeks–Chandler–Andersen (WCA) energies of the top molecule were 86.909 kJ/mol, -13.272 kJ/mol, 0 kJ/mol and 0 kJ/mol respectively.

**Table 8:** Binding energy and other energies values of **633972**.

S.N.	Type of energy	Value (kJ/mol)
1	van der Waal energy	-110.221
2	Electrostatic energy	-20.364
3	Polar solvation energy	86.909
4	SASA energy	-13.272
5	SAV energy	0.000
6	WCA energy	0.000
7	Binding energy	-56.947

## 4 Conclusion

In this present work, the authors consider the hetrocyclic molecules reported by the various research group with different biological activity. The top four molecules 633972, 633992, 8442220 and 8442281 are chosen based on the minimum binding energy of docking with ns2PB-nsP3 protease of DENV among the 138 molecules. These top four molecules are further refined on the basis of ADME properties and electronic descriptors. Based on the docking, ADME and DFT 633972 was chosen for the MD simulation and mmpbsa analysis. The RMSD from MD result clearly corroborates the docking result that the nsP2B-nsP3 protease of DENV gets stabilized after the binding with 633972. The binding energy value for the complex by mm-pbsa analysis was also found to be negative and support the inhibition.

## Declarations

### Author contribution statement

Prashant Singh - Conceived and designed the experiments; Wrote the paper.

Vijay Kumar - Performed the experiments; Analyzed and interpreted the data; Wrote the paper. Nidhi Shukla, Retu Shura, Kamlesh Kumari: Performed the experiments.

Rajan Patel: Contributed reagents, materials, analysis tools or data; Wrote the paper.

### Funding statement

This research did not receive any specific grant from funding agencies in the public, commercial, or not-for-profit sectors.

### Competing interest statement

The authors declare no conflict of interest.

### Additional information

No additional information is available for this paper.
